# Orthodontic management of Class III malocclusion in growing patients with RME and facemask: A case series

**DOI:** 10.6026/973206300220634

**Published:** 2026-02-28

**Authors:** Kasturi Mukherjee, Poulomi Roy, Amit Shaw, Amitava Bora, Prakash Banerjee, Aafreen Smriti Minz

**Affiliations:** 1 Department of Orthodontics and Dentofacial Orthopaedics, Burdwan Dental College and Hospital, Burdwan, West Bengal, India; 2 Department of Orthodontics and Dentofacial Orthopaedics, Dr R Ahmed Dental College and Hospital, Kolkata, West Bengal, India; 3 Department of Pediatric Dentistry, Burdwan Dental College and Hospital, Burdwan, West Bengal, India; 4 Intern, Kalinga Institute of Dental Sciences, KIIT Deemed to be University, Patia, Bhubaneswar, Odisha, India

**Keywords:** Facemask therapy, growth modification, malocclusion, maxillary deficiency

## Abstract

Orthopaedic treatment of skeletal class III malocclusion in children is critical because it can prevent potential surgical procedures. Initial management of class III malocclusion helps to avoid the harmful effects of facial deformity. Hence, we present four cases on the early orthopaedic therapy of class III malocclusion using rapid maxillary expansion (RME) and a face mask. All these cases presented with class III malocclusion, which included mid-face deficit and an anterior cross bite. All of them were treated with a combination of RME and facemask therapy. Combined skeletal and dental improvements resulted in satisfactory treatment of class III malocclusion.

## Background:

Skeletal class III malocclusion can arise due to undergrowth of the maxilla, overgrowth of the mandible, or both [1, 2-3]. In 25% of instances, there was maxillary skeletal retrusion and normal mandible sagittal connection, while 22% had both retrognathic maxilla and prognathic mandible [1]. In Europe, 1-5% of people Have class III malocclusion, while in Asia, it can reach 13% [4, 5]. Class III malocclusions pose a significant challenge for orthodontic treatment due to their high relapse rate. Treatment decisions for patients with skeletal class III malocclusions contain early growth modification, dental camouflage, and orthognathic surgery after growth has stopped. Orthopedic appliances, including protraction headgear and rapid maxillary expansion (RME), have been utilized to treat growing patients with early class III malocclusions [6]. An RME with a face mask can fix transverse and sagittal inconsistencies during the early phase of treatment [3]. This method is particularly effective in early mixed dentition, before maxillary and posterior sutures have closed. Therefore, it is of interest to discuss a series of cases with class III anterior cross bite were treated with rapid palatal expander and protraction headgear.

## Case 1:

**Figure 1:**
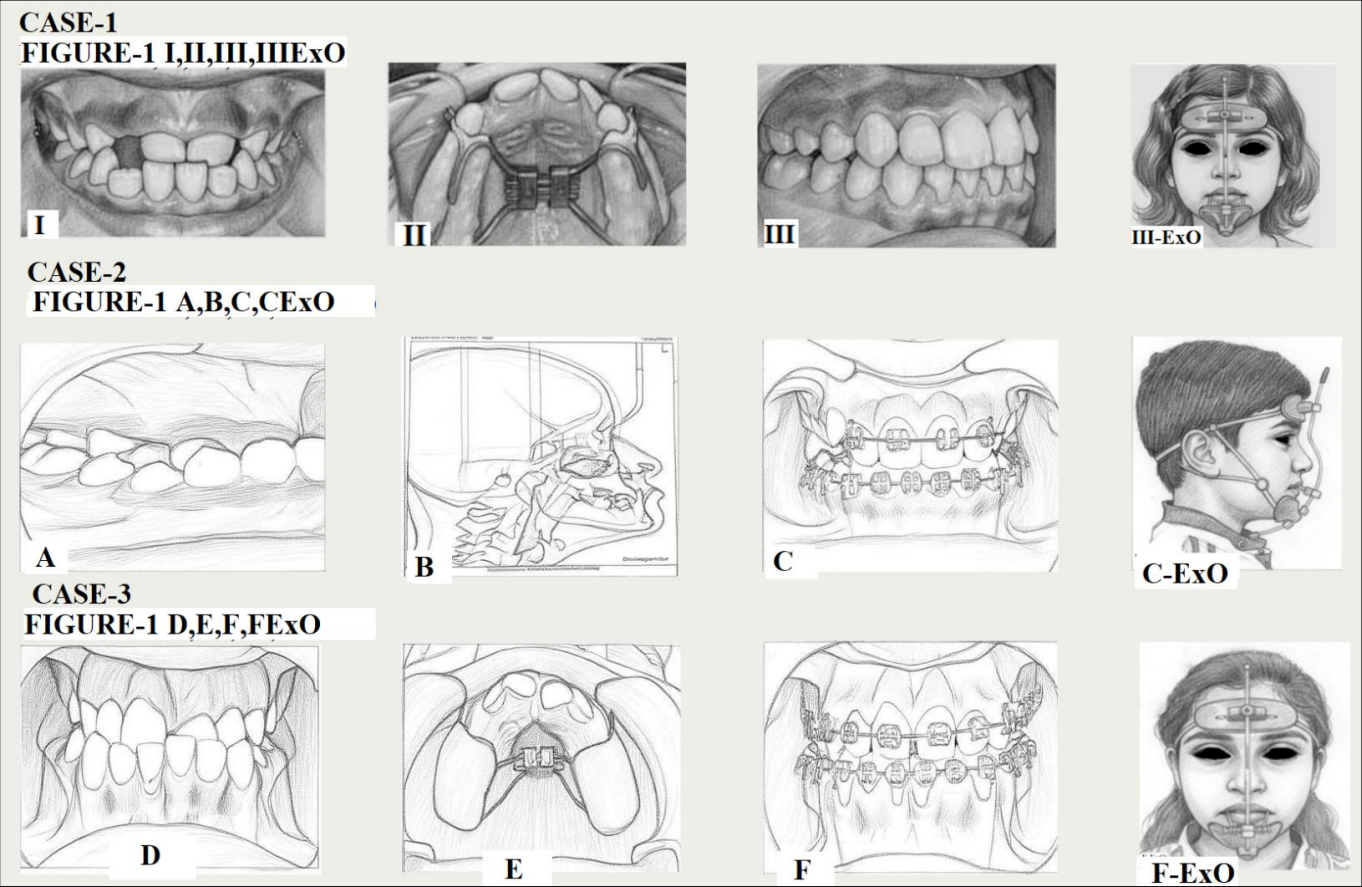
: I-Pre-treatment photographs, II-Intra oral expansion appliance (RME), III-Post treatment intra oral photo graph, III-ExO-Patient with Petit Facemask, A-Pre-treatment photographs, B- pretreatment lateral cephalogram, C-Fixed appliance therapy intra oral photo graph, C-ExO- Patient with Petit Facemask, D- Pre-treatment photographs, E- Intra oral expansion appliance (RME), F-Fixed appliance therapy intra oral photo graph, F-ExO- Patient with Petit Facemask.

A 7 year, old girl came with a problem of abnormal upper and lower anterior teeth. On extra oral examination, a concave profile was apparent with mild version of the lower lip. Intraoral examination revealed a mixed dentition stage, with permanent maxillary central incisors in reverse overjet relation revealing a reverse overbite of 3 mm. No premature incisal contact was seen and functional shift was not present (Figure 1I). Class III malocclusion was found in the family. The cephalometric evaluation shows a deficient SNA angle and increase in SNB angle.

## Case 2:

An 11-year-old male patient came with a main problem of backwardly placed upper front teeth (Figure 1A). Extra-oral evaluation showed that the patient had a symmetrical face with a mesoprosopic face form, concave profile and anterior divergence. The patient had competent lips with acute nasolabial angle and normal mentolabial sulcus. The smile of the patient was symmetrical and consonant. Intra-oral examination revealed a sufficient zone of attached gingiva with satisfactory gingival health. All permanent teeth had erupted other than third molars, and the upper canines are erupting on both sides. In the upper arch, proclined incisors were observed, along with anterior and posterior crossbite. Class III molar relationships were present on both sides on cephalometric radiograph (Figure 1B) and clinically. Overbite and negative overjet were 6 mm and 2 mm, respectively. A functional evaluation revealed a mature swallowing pattern, normal speech pattern, and ore-nasal breathing. There were no indications of temper-mandibular illness, and the mandibular closure path was normal. The case was treated with intra oral expansion appliance (RME) with face mask along with fixed orthodontic treatment. The treatment outcome was good as shown in Figure 1C.

## Case 3:

A 12-year-old girl came with mixed dentition, proclined upper and upright lower incisors, a prognathic jaw, a retrognathic maxilla, and Class III skeletal and dental malocclusion (Figure 1D). The patient's lips were competent, with a shallow mentolabial sulcus and a sharp nasolabial angle. The patient's smile was consonant and symmetrical. She had a decreased buccal overjet, anterior crossbite, and symmetrical maxillary mandibular arch forms with Class III relationships. Lower premolars were just half erupted. Patients were treated with intra oral expansion appliance (RME) (Figure 1E) and face mask along with fixed orthodontic treatment (Figure 1F). The outcome of the treatment was very good.

## Treatment plan:

It was planned to treat the three conditions having skeletal Class III with orthopedic appliances based on the clinical examination and cephalometric results. It was intended to use bonded HYRAX to expand the maxillary arch and a facemask to extend the maxilla (Figure 1II). Young Class III patients with maxillary deficit have been treated with a combination of maxillary protraction and fast maxillary growth (Figure 1III-ExO, Figure 1C-ExO, Figure 1F-ExO). It has been suggested that rapid maxillary expansion (RME) can disarticulate the maxilla from the surrounding bones that are joined by circum-maxillary sutures [7]. The second step of therapy involved using fixed orthodontic appliances for finishing.

For each of the three cases, there were two phases of treatment: Phase I: Orthopedic correction with RME and a facemask and Phase II: Dental correction and finishing and detailing (Figure 1III).

## Treatment progress:

## Phase I:

The patients received an appliance consisting of a bonded maxillary HYRAX screw and a protraction facemask. In order to engage the facemask elastics, appliance hooks were positioned on the buccal aspect next to the permanent canines in the expansion. For three weeks, a 90°C turn activation plan was followed twice a day once the expansion appliance was cemented. Following a week of fast palatal screw activation for maxillary expansion, the patient was recommended to wear a reverse pull-face mask on their chin and forehead. After two weeks of wearing 8 oz elastics, the weight was later increased to 14 oz. Each side of maxillary protraction requires 300-600g of effort. In order to achieve the greatest translator effect, elastics were positioned on the buccal surface of the first deciduous molar hook with a downward pull from the 30°C to 40°C occlusal plane. For six months, patients were told to wear elastic all the time, with the exception of school and outdoor sports (16 hours each day). Within six months, enough maxillary protraction was attained. For an additional three months, facemasks were employed for retention. The intra-oral and extra-oral photos show pre-operative and postoperative alterations.

## Phase II:

A 0.022" slot MBT prescription was used to begin post-orthopedic traction fixed orthodontic treatment. Niti wires measuring 0.014 x 0.016 x 0.017 x 0.025 were used for initial leveling and alignment in all non-extraction situations. Stainless steel wires measuring 0.017 x 0.025 and 0.019 x 0.025 were then used. The course of treatment lasted almost two years in total.

## Treatment results:

In the final stage of orthodontic treatment showed improvement in facial profile in all the four cases. Well-aligned dental arches, normal overjet, overbite, and matching midlines were obtained.

## Discussion:

Class III malocclusion, which can leads to both functional and aesthetic difficulties, can be caused by skeletal or dental abnormalities. RME and facemask therapy are the most widely used orthopaedic treatment regimens for class III malocclusion [8]. Both the maxillary and mandibular components are affected simultaneously by this treatment. The best time to use an orthopaedic method to treat class III malocclusion is during either the prepubescent or pubertal stages of growth and development [9]. Maxillary protraction has a reported success rate of 66-75% and is typically regarded as stable [10, 11-12]. Bengal *et al.* successfully performed distraction osteogenesis in the premaxilla using a bite block-style device with a Hyrax screw positioned parallel to the mid-palatal suture [13]. It is widely acknowledged that mid-face deficient class III patients should receive treatment before the age of 7-8 years [10]. Even in individuals who do not require an increase in transverse dimension, it is recommended that the appliance be engaged for 8-10 days prior to facemask insertion [12]. According to Turley [14], fast palatal expansion shows favorable reaction. Protraction headgear and palatal expansion appliances have comparable effects on these sutures. For example, the zygomatic buttress, particularly the zygomaticomaxillary suture, has been identified as a key barrier to forces generated by both palatal expansion and maxillary protraction. As a result, many clinicians recommend maxillary expansion a week before beginning facemask therapy. Even in the absence of maxillary constriction, crowding, or posterior crossbite. As a result, in all the four cases, we used bonded palatal extension for just 10-20days since it interfere the maxillary suture system and increases maxillary protraction. This bonded quick palatal expansion device also inhibits undesired tooth movements and did not cause overcorrection or scissor bite in the cases.

The facemask therapy effects include skeletal and dental alterations to the maxilla and mandible [15]. The force on the maxilla, shift it downward and forward. As a result of this impact, the mandible rotated downward and backward, enhancing the sagittal connection between the maxilla and mandibular bones. However, this resulted in an increase in the lower face height. Because the patient's mandibular plane is relatively low to normal, the effect was aesthetic. Anterior overjet was significantly improved by this jaw movement [16]. Dentally, the protractive force led the upper incisors to procline, whereas the force of the chin cup caused the lower incisors to retrocline. Rutili *et al.* found in their prospective long-term study of the effects of maxillary expansion during facemask therapy that the average anterior movement of point Maxillary teeth migrated 2.73 mm, while a post treatment was 1.54 mm [10]. They asserted that both orthopedic and dental aspects contributed to the advantageous overjet. Furthermore, they stated that while there were few statistically considerable alterations in the jaw and its dentition, such adjustments helped to rectify Class III. In our patients, the horizontal changes in point A of the maxilla after protraction were similar. Since the overjet correction needed was minimal and the incisors' change in inclination was corrected during the fixed appliance therapy phase, the patient's maxillary incisors displayed mild retroclination rather than proclination in comparison to treatment results from other studies [10- 11]. This resulted in improved lip posture and a straighter skeletal and soft tissue profile. According to an in vitro investigation, the greatest translatory impact was obtained when the facemask was pulled downward from 45°C to 30°C [17]. We preferred a 30°C angulation to generate a satisfactory clinical response, which is comparable to the study of Ngan *et al.* [11] a more aesthetically attractive smile resulted from the maxilla's downward migration, which improved the exposure of the upper incisor. A systematic meta- analysis found that dental side effects were more noticeable when no expansion was performed, despite some recent research suggesting that transverse expansion does not have a major impact on sagittal maxillary development by a facemask [18, 19]. Additionally, they claimed that although the more recent idea of alt-RAMEC (alternating RME and contraction) improved face mask treatment, more randomised controlled trials were required [19]. Hiremath *et al.* [20] demonstrated that treated patients with a maxillary deficit but normal mandibular proportions often showed acceptable stability, despite concerns about the stability of Class III orthopaedic treatment. Furthermore, it has been demonstrated that the duration of stabilisation is inversely connected with the severity of relapse [21]. Kumar *et al.* stated that, RME can be used as treatment choice to treat skeletal Class III malocclusion patients [22]. According to Jha & Chandra, initial mixed dentition period is the good time for class III treatment [21]. Rapid maxillary expansion (RME) and a facemask (FM) are a well-established orthopaedic protocol for managing Class III malocclusion in growing patients. This protocol has seen significant advancements in knowledge, especially in the use of skeletal anchorage and altered expansion protocols (Alt-RAMEC) to maximize skeletal effects over dental compensation.

## Conclusion:

Facemask therapy is an effective early therapy for class III malocclusion in growing patients. The maxilla's forward displacement and the mandible's downward, backward rotation are the primary means of profile correction. When a reverse pull face mask is worn, the gonial and articular angles open up further, the mandibular plane angle steepens and the height of the lower anterior face increases. Early correction of crossbite linked to class III malocclusion is necessary to avoid negative effects on maxillary growth.

## Clinical significance:

Early intervention is crucial for treating class III malocclusion in growing patients. The uncertainty of long-term stability has led to extensive research on this topic. Early-stage treatment options include intraoral and extraoral appliances. RME and protraction face masks are beneficial for treating early mixed dentition.
